# 

**Published:** 1918-05-11

**Authors:** 


### CONTENTS.

PAGE

**Civil Hospitals and the Care of Military Patients: Some United States' Problems** 109

**Hospital and Institutional News** ... .. .. 111
Our Brothers of the United States—Special Rations Scales for Hospitals—Hospitals and Their Milk Supply—"Earlsmead" Home of Recovery—A Historical Document—King Edward Memorial Hospital, Birmingham—The Anatomical Society of Great Britain and Ireland—Vaccination of Children in Poor-Law Institutions—The Beginning of the Saturday Fund—A Bedroom Bazaar—The Woman House Surgeon—A Request and a Gift to Portsmouth Hospital—A Neglected Source of Income—A Double Gift—Legacies and Maintenance—Staff Changes and Annuities at Nottingham—Doctors' Certificates—The Health of London School Children—The War and Infant Mortality—Municipalising the Nurseries—This Week's Drug Market—To Our Readers.

**A New Disease or Food Poisoning?** ... ... 115

The Position Defined and the Public Directed.

**The Position of Science in Education** ... ... 117
Report of the Prime Minister's Committee.

**Defects in the Lunacy Law** ... ... ... 119

Some Changes held to be Desirable.

**The Organisation of British Ophthalmologists** 120
A Representative Council Formed.

**The Adventures of the Blind** ... ... ... 121

The Doings of St. Dunstan's Men.

**King's Bench Judges at Tea** ... ... ... 122

Tea Not a Food but By Order.

**The Matrons' and Sisters' Department** ... 123
XIII. Nursing Progress and its Developments.
Round the Hospitals.
Splendid College Reports—A Great Opportunity for College Members—Hospitality and a Conference—The M.P. and Enemy Tarradilloes—The V.A.D. Recruiting Problem—A Ready Reckoner for War Hospitals.

**The College of Nursing (Limited by Guarantee)** 127

Booming Ahead!

**The Ed tor's Letter-Box** ... ... ... ... 129

**Nurses' Tribute Fund** ... ... ... ... 130

**[illegible] Institutional Worker** ... .. (Suppl.) 1

